# Relaxation Techniques for People with Chronic Obstructive Pulmonary Disease: A Systematic Review and a Meta-Analysis

**DOI:** 10.1155/2015/628365

**Published:** 2015-08-03

**Authors:** Eleonora Volpato, Paolo Banfi, Sheena Michelle Rogers, Francesco Pagnini

**Affiliations:** ^1^Department of Psychology, Università Cattolica del Sacro Cuore, 20123 Milan, Italy; ^2^Department of Neuromuscular Disease, Fondazione Don Carlo Gnocchi, 20149 Milan, Italy; ^3^Dartmouth College, Hanover, NH 03755, USA; ^4^Niguarda Ca' Granda Hospital, 20162 Milan, Italy

## Abstract

*Introduction*. Chronic Obstructive Pulmonary Disease (COPD) people suffer from severe physical impairments, which often elicit significant psychological distress and impact their quality of life. This meta-analysis aimed to assess evidence from the scientific literature on the effects of relaxation techniques.* Methods*. We investigated 9 databases to select 25 RCTs. Studies included both inpatients and outpatients with COPD. Both respiratory and psychological outcomes were considered.* Results*. Relaxation techniques showed a little positive effect on the value of the percentage of predicted FEV_1_ (*d* = 0.20; 95% Cl: 0.40–−0.01) as well as a slight effect on levels of both the anxiety (*d* = 0.26; 95% Cl: 0.42–0.10) and depression (*d* = 0.33; 95% Cl: 0.53–0.13). The higher effect size was found in the quality of life value (*d* = 0.38; 95% Cl: 0.51–0.24). The assessed quality of the studies, based on the PEDro Scale, was generally medium/high.* Conclusion*. Relaxation training can have a moderate impact on both psychological well-being and respiratory function, resulting in noticeable improvements in both. Although higher quality research is required, our results sustain the importance of relaxation techniques as a tool to manage COPD.

## 1. Introduction

The Global Initiative for Chronic Obstructive Lung Disease (GOLD) defined Chronic Obstructive Pulmonary Disease (COPD) as airflow limitation that tends to not be fully reversible and which is usually both progressive and associated with an abnormal inflammatory response of the lungs to noxious particles or gases [1]. It is predicted that the burden of COPD will be even more apparent in the coming decades, due to the continuous exposure to risk factors as well as the increase in life-expectancy [2, 3]. It is expected that COPD will move from the sixth to the fourth cause of mortality and morbidity in the world [4]. Moreover, this illness accounts for significant health-care costs worldwide [5], prevailing both in developed and in developing countries [2]. It is important to note that COPD severity is related to a worse health-related quality of life [6], which is also characterized by a worsening in emotional well-being, shown to be related to fatigue and other coping strategies of everyday life [7]. The psychological distress of these patients is often characterized by both anxiety and depression [8] as well as by reported feelings of helplessness, powerlessness, loss of mobility and freedom, tense relationships, panic attacks, and growing social isolation in patients' narrations [9–11]. Furthermore, the cognitive profiles of these patients are compromised by hypoxemia, hypercapnia [12], and sleep problems, caused by coughing and breathlessness [13, 14]. State-of the-art research methods allow us to highlight how, despite the fact that patients with COPD often exhibit symptoms of psychological distress, interventions dedicated to them tend to pay attention mostly to the physical aspects of the disease, with a considerable waste of resources [15]. However, it could be important to consider also the psychological aspects, because they can have significant impacts on both the quality of life of individuals and on the therapeutic relationship. This also might be a way to check if the active management of the disease also from a psychological point of view can improve outcomes and reduce the waste of material and social resources [16]. In this perspective, relaxation techniques are often used to inhibit anxiety, increasing the patient's perception of self-control or modulating his or her emotions, and in order to promote the perceived well-being of the subject. However, the effectiveness of these techniques to reduce COPD patients' symptoms is not always clear, since studies are characterized by different methodological quality rendering the results often inconsistent. Indeed, many studies showed improvements in oxygen saturation during the use of a relaxation method, such as guided imagery, even in patients with COPD [17, 18]. Moreover, even if some researchers discovered that methods such as progressive muscle relaxation can reduce psychological distress in patients with COPD [19–21], the cost-effectiveness of adding these techniques to a rehabilitation program is not always clear [21, 22]. It is also important to note that drug treatments are not always effective in providing a certain level of relief in case of dyspnea, especially in cases where the causes of the disorder are unclear [23]. Due to this fact, some authors have evaluated the safety, feasibility, and effectiveness in reducing dyspnea and anxiety of programs based on relaxation trainings, such as yoga. Results revealed that these programs were indeed effective and also resulted in better functioning in daily life. Moreover, they detect a distress decrease and then a better functioning in daily life [24]. Likewise, other studies stressed the effectiveness of yoga in relieving levels of anxiety and depression, increasing sense of control and self-esteem, and instilling hope in patients [25–27]. Other techniques, such as Tai Chi [28, 29], biofeedback, and breathing control, were used, resulting in an improvement in breathing capacity and function of the extremities and increasing the strength of muscles important for respiration [30–32]. Finally, other previous studies stressed the importance of the application of methods such as distraction therapy [33–35] or acupressure [36], since they have been proven to improve psychological well-being and physiological parameters.

It is important to note that there is a lack of literature providing evidence about the efficacy of relaxation techniques in COPD patients both in terms of practicability and feasibility in everyday life and in terms of health improvements. There are not clear data about immediate and long term effects of these techniques.

Given this lack of knowledge, we focused our meta-analysis on the effectiveness of relaxation techniques on COPD patients. Our goal is to summarize and evaluate existing evidence of studies concerning the effects of such interventions so that health professionals can adopt and integrate them to improve the quality of life of their COPD patients. In particular, we derived some following hypotheses from the literature.

(i) Relaxation techniques can be effective in training patients with COPD, but only under certain conditions.
(ii) There is a relationship between the use of relaxation techniques and the reduction of anxiety and depression, as well as the improvement of quality of life and the percentage of Forced Expiratory Volume in the First Second (FEV_1_).

## 2. Method

### 2.1. Literature Search Strategy

The main aim of the study selection is to examine the levels of anxiety, depression, quality of life (QoL), and percentage of predicted FEV_1_ of inherent value both before and after treatment.

We have referred to the following research computer databases: PsycINFO, PubMed, Scopus, Web of Science, MEDLINE, Cochrane, PsycARTICLES, SpringerLink, and ClinicalTrials.gov. Unpublished studies were not considered.

The search strategy used a combination of the following words, searched as title, key words, abstract, and MeSH subjects heading terms: “relaxation,” “relaxation training,” “relaxation technique,” “relaxation therapy,” “progressive muscle relaxation,” “progressive relaxation,” “meditation,” “guided imagery,” “distraction therapy,” and “biofeedback”, each of them together with the term “copd” by the Boolean operator “AND.” Moreover, references cited in the research studies were gathered and recent reviews were scanned for further trials, using “cited by” search tool. In addition, authors who discussed this topic in the past were contacted in order to gather more data and information about their studies. We have selected only articles published in English between 1970 and 2015 and studies performed only on human adults.

### 2.2. Inclusion and Exclusion Criteria

Before implementing the literature review, we defined the inclusion and exclusion criteria (Table 1). We included only Randomized Controlled Trials both prospective and single or double blinds. Studies needed to include subjects both hospitalized and outpatients, who were affected by COPD at varying levels of severity and who had very severe airflow obstruction. Studies that included subjects who did not have this diagnosis were not accepted even if they were in the control group to insure that both groups were comprised of subjects similar in sociodemographic and clinic characteristics in both groups. We have also excluded studies in which the treatment provided for comparison between a control group and one subjected to a pulmonary rehabilitation was a relaxation component that constituted only a few minutes, or studies that compare two rehabilitation programs in which the relaxation is present in equivalent terms. We have also required that the control group employ a usual rehabilitation treatment or a placebo, which must consist of activities that are not truly relaxing (i.e., home crafts). As we have already stated, however, studies had to detect at least one of the variables considered moderators of the effect, that is, anxiety, depression, quality of life, or percentage of predicted FEV_1_ of inherent value. Furthermore, in order to make the effect size computation possible, only studies that reported sufficient communication of the results were included (e.g., mean and standard deviation). Moreover, studies had to investigate the effects of one or more relaxation trainings, such as relaxation techniques, progressive muscle relaxation, guided imagery, distraction therapy, biofeedback, breathing techniques (diaphragmatic breathing and Pursed-Lips Breathing), yoga, or Tai Chi.

### 2.3. Study Selection

**Figure 1:**
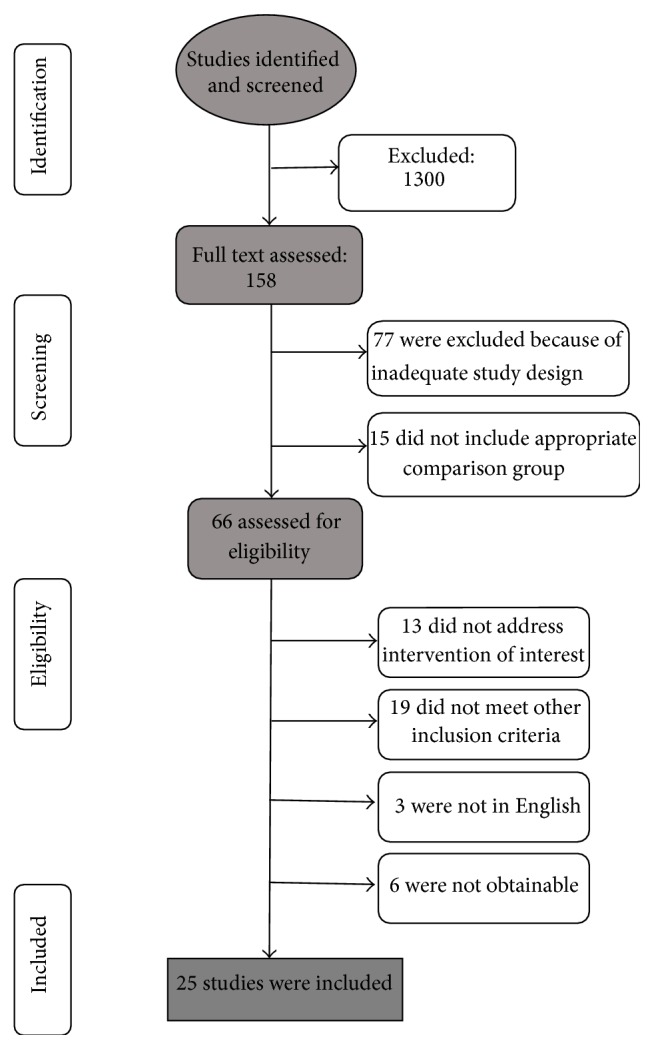
Flow chart of study selection.

We identified the potential articles and read the abstracts to determine whether they met the inclusion criteria. We excluded 77 studies, because they were case reports, letters, reviews, editorials, or cohort studies (see Figure 1). For the remaining papers, we read the full text: in this way, an additional 15 studies that did not include an appropriate comparison group were excluded (see Figure 1). Though consistent with our inclusion and exclusion criteria, another 13 studies were excluded, because they adopted a different intervention such as Inspiratory Muscle Training (IMT), autogenic drainage, psychoeducational care, or active cycle breathing techniques (ACBT). After the exclusion of these 28 studies, an additional 3 studies were excluded because they were not in English, 6 were excluded because they were not obtainable, and 19 were excluded because they did not meet one of the inclusion criteria shown above. The resulting meta-analysis included 25 studies (see Figure 1). Only one of the studies included is under submission [55] while 24 are published in a scientific journal.

### 2.4. Data Extraction and Coding

According to the above criteria, we extracted data related to the variables considered potential predictors of study results, which included anxiety, depression, quality of life, or percentage of predicted FEV_1_ of inherent value, examined before and after relaxation training. It is important to note that, in this meta-analysis, the coding system of studies was configured as directed and was based on a careful reading of the articles. We have also codified data on study design, year, geographic origin of the study, number of subjects and number of males and females, number of subjects per group, patients diagnosis, mean age, intervention characteristics (type of relaxation training, duration, number of sessions, concurrent therapies, trial context, and homework), and assessment measures. Basic descriptive information about the statistical procedures used in the research was also annotated. Finally, where possible, the number of cases of failures and/or retirements and discontinuities to treatment was reported.

### 2.5. Risk of Biases Assessment

**Figure 2:**
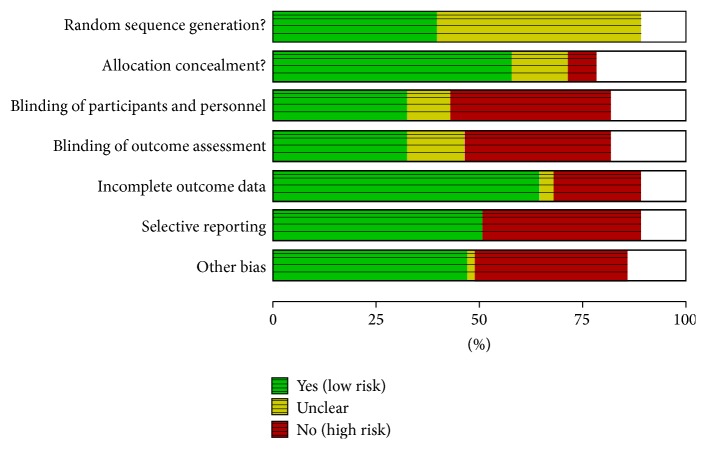
Risk of bias graph: judgments about each risk of bias item presented as percentages across all included studies.

**Figure 3:**
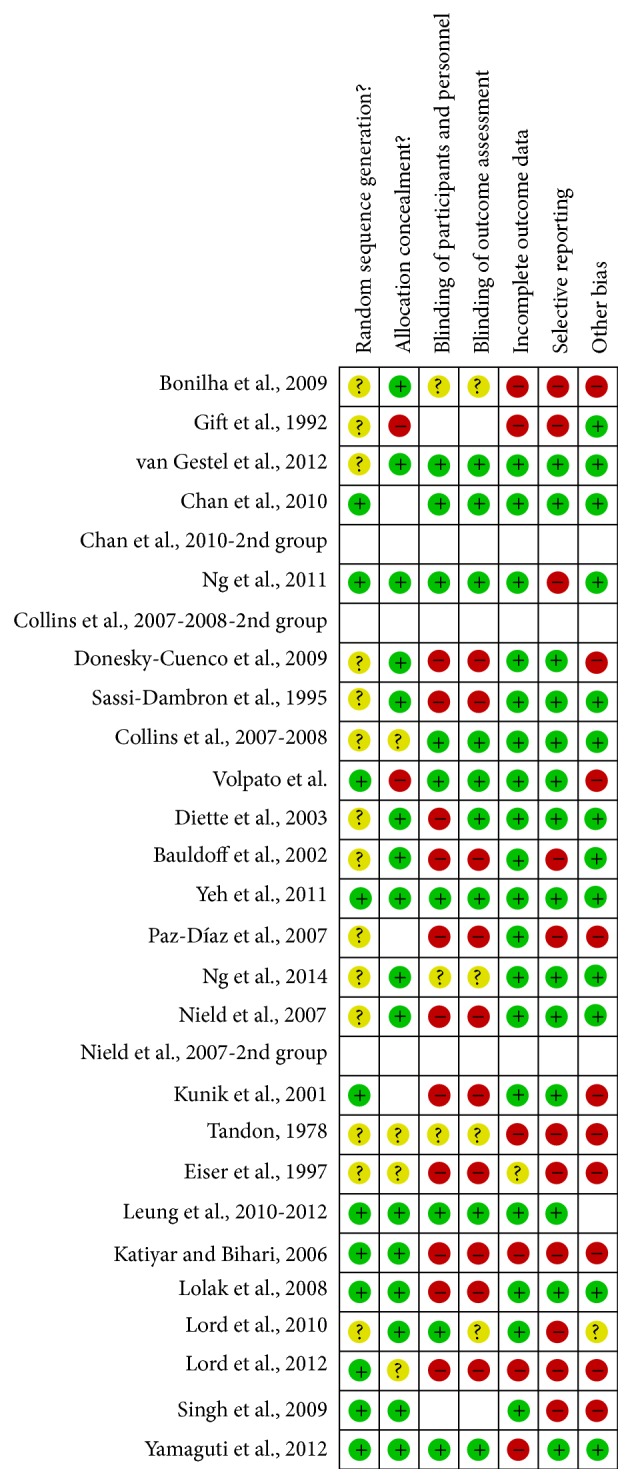
Risk of bias summary: review authors' judgements about each risk of bias item for each included study.

Two authors evaluated the risk of biases independently. For this purpose, the Assessment Tool by Cochrane Collaboration was used. It consists of seven items regarding the selection, performance, detection, attrition, reporting, and other sources of bias [56]. The two authors mentioned previously discussed the incongruities with the corresponding author (Figures 2 and 3). We used funnel plots to check for the existence of publication bias (Figures 5, 7, 9, and 11). Finally, we performed forest plots (Figures 6, 8, 10, and 12) by using Review Manager Software 5.3 (Cochrane Collaboration).

### 2.6. Methodological Quality

We have also assessed the methodological quality of the RCTs included in the meta-analysis (Table 2). For this purpose, as appropriate by studies design, we used the PEDro Scale, which is based on the Delphi List, developed by Verhagen et al. to evaluate the RCTs quality [57]. Two researchers evaluated the quality of each study, independently. Each paper received a one point on each satisfied item (except the first item) out of the total score of the PEDro Scale (range of 0–10). Also in this specific case, they discussed the discrepancies with the corresponding author.

### 2.7. Data Analysis

Data analysis was performed using the statistical software Statistical Package for Social Science (SPSS), Version 20. All data extracted were reported as they were given in the publication.

The effect sizes or size effects were calculated for each study, in relation to the variables considered. They were calculated between both groups at “Time 2,” commensurate to an average of 8–12 weeks. These values were computed using Cohen's *d*, which allows the determination of the overlap between the distributions of the experimental group and the control group. When the necessary data were available to estimate the standardized difference between the means of the groups (e.g., mean and standard deviation), we applied them to the following formula: *d* = (*M*1 − *M*2)/*S* [58]. *M*1 is the mean of the experimental group, *M*2 is the mean of the control group, and *S* is the standard deviation of the general sample, which is computed with the formula: $S=\sqrt{\left(\left(n_{1}-1\right)s_{1}^{2}+(n_{2}-1)s_{2}^{2}\right)/\left(n_{1}+n_{2}-2\right)}$. When the necessary data was not available to calculate effect sizes using this formula, we proceeded using other expressions and then converted them into *d* through appropriate equations [59, 60]. These effect sizes were interpretable in terms of Cohen's convention, whereby 0.2 is small, 0.5 is medium, and 0.8 is large [61]. Moreover, for each study, we calculated the effect size introducing a correction term capable of producing an undistorted standardized difference between means, based on Hedges' formula [62]. Furthermore, because the effect is indicative of the intensity of the relationship between independent and dependent variables, the effect's convertibility measured as the difference between averages becomes relevant, also in terms of correlation: *r* = *d*/√(4 + *d* ^2^) [60]. After calculating the effect sizes for each study included, we calculated the index medium that expresses the extent of the overall effect. Later, we calculated the limits of the confidence interval around the mean value found, as well as the average of the effects from *r*, using the appropriate formulas [60]. We also tested the invariance between studies considered in the meta-analysis, designed to examine whether the effects share a common effect size or if the variability requires clarification from the input variables from which the effects are taken [63]. In order to assess the relationship between predictors and most effect sizes considered, we performed multiple regressions, weighted by the reciprocal of the variance of the same effects. However, we calculated the fail-safe number, which is an index regarding the valuation of stability analysis carried out and the Binomial Effect Size Display (BESD), able to interpret the indices of effect size [64]. Heterogeneity was evaluated via the chi-square test. Finally, it should be noted that, where possible, significance levels, effects sizes, odds ratio, and 95% Cls were calculated.

## 3. Results

### 3.1. Description of Studies

We reviewed 158 full text papers and 25 of them met the inclusion criteria (see Figure 1). We included only RCTs, 5 of which were prospective RCTs; 2 RCTs used a block randomization and 4 were single blind. They involved only people with COPD, mainly moderate to severe; one study considered COPD and asthma patients with Flexible Bronchoscopy (FB) and another one included patients with severe airflow obstruction, with or without emphysema. As regards the publication's year, four are dated 1978, 1992, 1995, and 1997, respectively, while the others are all dated from 2000 to 2015. The countries in which the studies were conducted can be broken down into the following: the United States of America (44%), China (12%), the United Kingdom (12%), Brazil (8%), Australia (8%), India (8%), Germany (4%), and Italy (4%). The relaxation methods included were the following: progressive muscle relaxation (PMR), breathing techniques (retrained or rhythmic breathing and Pursed-Lip Breathing (PLB), diaphragmatic breathing (DB)), distraction therapy, yoga, Tai Chi, and biofeedback (Respiratory Biofeedback Training (RBF) or Ventilation Biofeedback Training (VBT)). 12 of these studies (48%) included a combination of these techniques (e.g., breathing techniques and relaxation, etc.) (Table 2). In 10 studies (44%), patients underwent private relaxation training, while 14 studies (56%) involved training in small groups. Moreover, in 10 cases (40%) patients were required to do exercises at home that could further enhance the effectiveness of the techniques implemented, while in 13 studies (52%) they were not required to. In 2 cases (8.3%) it was not specified. In 3 studies, there were three groups, the third of which was subjected to another treatment or to the experimental treatment combined with other activities.

In case of multiple treatments reported in the same paper, each group was considered separately and compared with a control group.

As we have already specified, the comparison condition of the control studies consisted of a usual rehabilitation, laying down, placebo treatments, or activities that were not particularly restful (i.e., handcraft works).

All studies met the inclusion criteria and therefore detected one or more of the variables considered the predictors of the effect (FEV_1_, anxiety, depression, and quality of life). The main assessment measures used in the studies included one or more of the following: State-Trait Anxiety Inventory (STAI/SSAI) (8 studies, 32%), Hospital Anxiety and Depression Scale (HADS) (5 studies, 20%), Centre of Epidemiologic Studies of Depression Scale (CES-D) (4 studies, 16%), Beck Depression Inventory (BDI) (1 study, 4%) and Geriatric Depression Scale (GDS) (1 study, 4%), and Beck Anxiety Inventory (BAI) (1 study, 4%), which assessed anxiety and/or depression. Instead, Chronic Respiratory Questionnaire (CRQ) (6 studies, 24%), Saint George's Respiratory Questionnaire (SGRQ) (9 studies, 36%), Quality of Well-Being (QWB) (1 study, 4%), Visual Analog Scale of QoL (1 study, 4%), and MOS Short Form-36 Health Survey (SF-36) (6 studies, 24%) were used to measure the quality of life (Table 2). A pulmonary function test was generally adopted to assess the percentage of predicted FEV_1_ of inherent value and only one study exclusively measured this value (1 study, 4.16%).

The pooled sample consisted of 1426 subjects (mean = 57.31; SD = 48.461; range = 10–206), in which 615 (mean = 26.3; SD = 21.648; range = 5–94) were allocated for the experimental group, 627 (mean = 24,88; SD = 20,352; range = 5–98) were assigned for the control group, and, finally, 102 (mean = 3.92; SD = 14.043; range = 0–69) were for a third group. The mean age of the subjects was 67,12 (SD = 8,09).

Dropout rate ranged from 0 to 48 subjects (12%). The reasons for these dropouts were not always reported. However, in these cases, it was indicated that the patient had either died, experienced a worsening in his or her clinical condition, or discontinued treatment due to a lack of motivation. Follow-up studies were done only in a few of these cases.

Finally, regarding the statistical analysis generally used, the analyses run on the 25 included studies, in addition to detecting the frequency characteristics and descriptive statistics, were the following: *t*-test for independent samples or paired samples, one-way ANOVA (analysis of variance), factorial ANOVA, ANCOVA (analysis of covariance), and MANOVA (multivariate analysis of variance). Rarely, the nonparametric Wilcoxon signed-rank test and the Mann-Whitney *U* test were applied (Table 2).

### 3.2. Overall Effect Sizes

Table 3 shows the effect sizes for each study, considering the principle effect's moderators.

The average effect, calculated considering what the “Time 2” of treatments (corresponding to an average of 8–12 weeks) is on all trials and all variables considered moderators of the effect, is 0.31 (95% Cl: 0.39–0.23). According to Cohen's conventional criteria [61], this effect turns out to have little significance and it is positive.

The effect size relating to the value of the percentage of predicted FEV_1_ is 0.20 (95% Cl: 0.40–0.00), indicating a slight positive effect.

The effect of the studies with respect to the moderator variable “anxiety” had a value of 0.26 (95% Cl: 0.42–0.10), thus showing a small positive effect size. The effect size of the moderator variable “depression” at the end of the intervention is 0.33 (95% Cl: 0.53–0.13), indicating another little positive effect.

Finally, the effect size of the moderator variable “quality of life” had a value of 0.38 (95% Cl: 0.51–0.24), at the “end of treatments,” being also positive and small. The Binomial Effect Size Display (BESD), pertaining to the overall average effect, allows us to infer a moderator variables' improvement of 63% with regard to the experimental group and of 37% for the control group (“Time 2”).

### 3.3. Effect Size by Relaxation Techniques Implementation and Types

**Figure 4:**
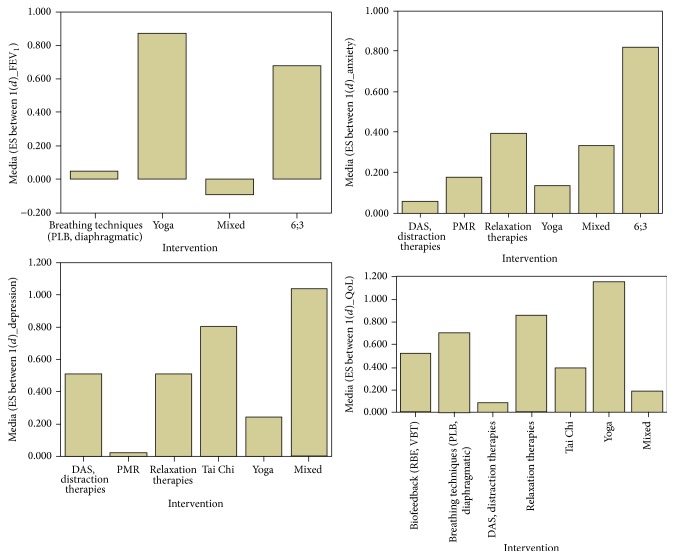
Effect sizes in relation to the intervention proposed in the studies included. DAS: Distractive Auditory Stimuli; 6;3: relaxation therapies and breathing techniques; mixed: many relaxation techniques combined together in the same session.

**Figure 5:**
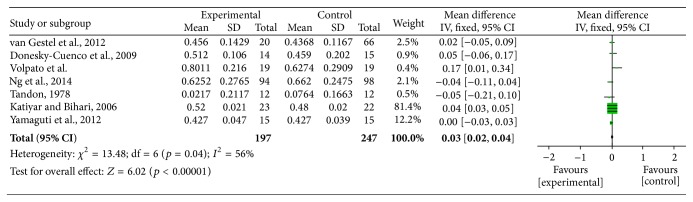
Forest plot of comparison, outcome: FEV_1_.

**Figure 6:**
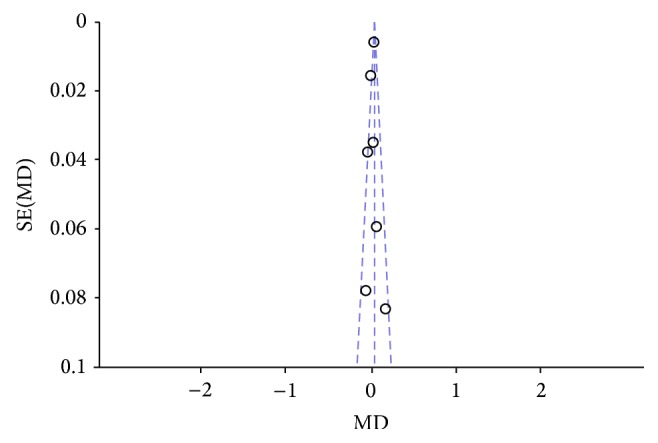
Funnel plot of comparison, outcome: FEV_1_.

**Figure 7:**
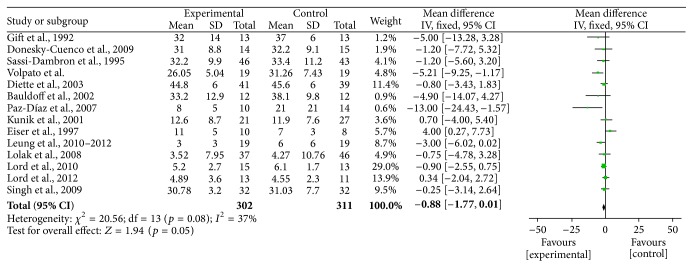
Forest plot of comparison, outcome: anxiety.

**Figure 8:**
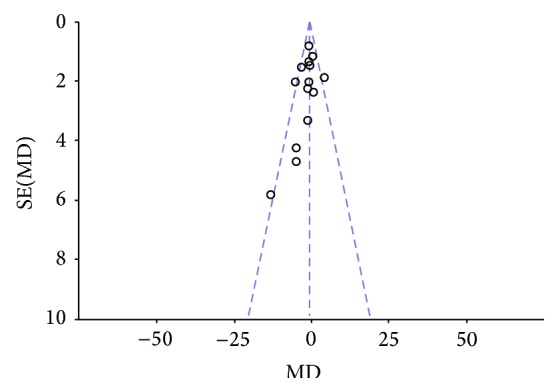
Funnel plots of comparison, outcome: anxiety.

**Figure 9:**
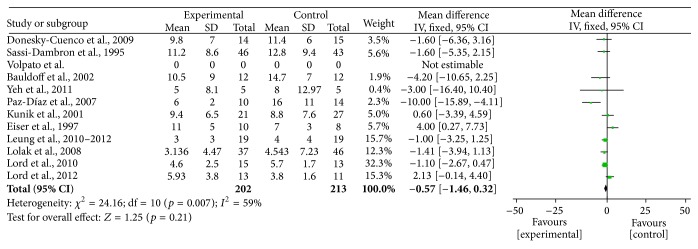
Forest plot of comparison, outcome: depression.

**Figure 10:**
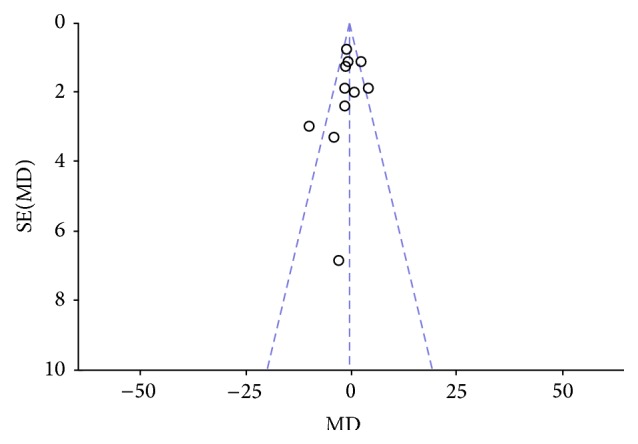
Funnel plot of comparison, outcome: depression.

**Figure 11:**
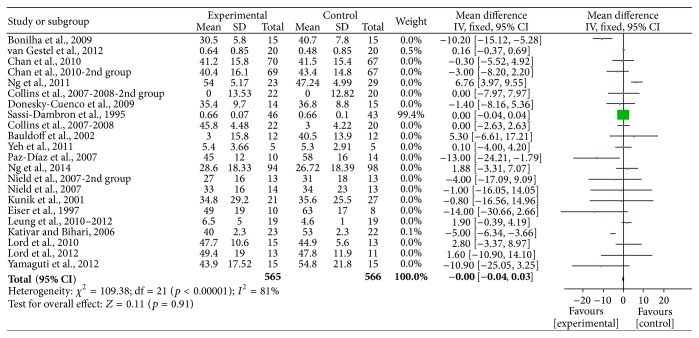
Forest plot of comparison, outcome: quality of life.

**Figure 12:**
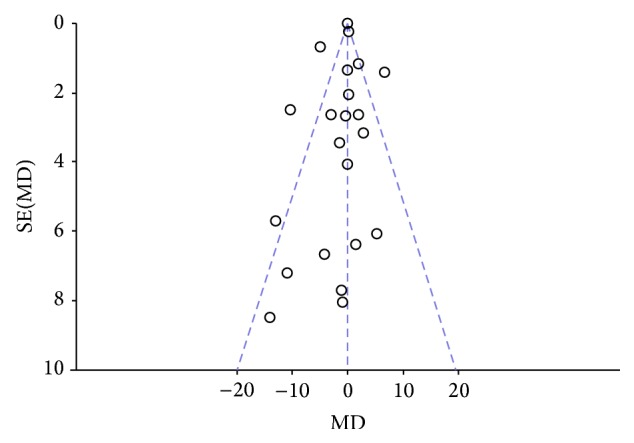
Funnel plot of comparison, outcome: quality of life.

The kind of* intervention* seems to influence the efficacy of the treatment (Figure 4). In particular, there are significant differences in the FEV_1_ (*F*(3) = 34.242; *p* = 0.000  *p* < 0.05; *η* = 0.530; Observed Potential = 1.000), comparing cases in which the breathing techniques were used and those in which a combination of relaxation techniques was used (*p* = 0.000 (95% Cl.: −0.101–−0.179)). There are also differences in the FEV_1_ comparing cases in which a combination of many relaxation techniques was used and those in which yoga was used (*p* = 0.000 (95% Cl.: 0.439–1.527)) and also comparing cases in which yoga and breathing techniques were used (*p* = 0.001 (95% Cl.: 0.299–1.385)). Finally, there is a significant difference between a combination of many relaxation techniques and the adoption of the combination of relaxation therapies and the breathing techniques (*p* = 0.000 (95% Cl.: −0.808–−0.729)).

Similarly, there are significant differences in the* anxiety* (*F*(5) = 7.176; *p* = 0.000  *p* < 0.05; *η* = 0.199; Observed Potential = 0.999), depending on the type of intervention adopted. The main differences are between the combination of relaxation therapies and breathing techniques and the implementation of many relaxation techniques (*p* = 0.000 (95% Cl.: −0.640–−0.328)) and between this second option and yoga (*p* = 0.003 (95% Cl.: 0.46–0.358)).

Considering* depression*, there are differences (*F*(5) = 6.022; *p* = 0.000; *η* = 0.241; Observed Potential = 0.993) between the adoption of a combination of techniques and progressive muscle relaxation (*p* = 0.000 (95% Cl.: 0.209–0.546)) or Tai Chi (*p* = 0.001 (95% Cl.: 0.572–0.235)).

Finally, in regard to the* quality of life*, differences (*F*(6) = 13.292; *p* = 0.000; *η* = 0.273; Observed Potential = 1.000) are between a combination of techniques and biofeedback (*p* = 0.000 (95% Cl.: 0.41–−0.26)) or breathing techniques (*p* = 0.038 (95% Cl.: −0.96–−0.01)), Distractive Therapies (*p* = 0.001 (95% Cl.: 0.02–−0.17)), and relaxation therapies only (*p* = 0.035 (95% Cl.: −1.31–−0.03)). Other significant differences are between the implementation of Distractive Therapies and breathing techniques (*p* = 0.010 (95% Cl.: 0.11–1.05)), relaxation therapies (*p* = 0.013 (95% Cl.: −1.40–−0.013)), Tai Chi (*p* = 0.030 (95% Cl.: −0.61–−0.17)), and yoga (*p* = 0.043 (95% Cl.: 0.02–2.12)).

Doing or not doing the* homework* was a factor that predicted a significantly different effect between groups; indeed, doing the homework improves* anxiety* (*F*(2) = 12,041; *p* = 0,000  *p* < 0,05; *η* = 0.141; Observed Potential = 0.995; *M* = 0.463 SD = 0.446 (yes homework); *M* = 0.190; SD = 0.348 (no homework)) and* depression* (*F*(1) = 6.991; *p* = 0.010  *p* < 0.05; *η* = 0.066; Observed Potential = 0.745; *M* = 0.432; SD = 0.376 (yes homework); *M* = 0.246 SD = 0.331 (no homework)).

Varying* the implementation* of the relaxation technique (individual versus group) shows a significantly different effect on* anxiety* (*F*(1) = 18.242; *p* = 0.000  *p* < 0.05; *η* = 0.110; Observed Potential = 0.989; *M* = 0.454; SD = (group); *M* = 0.176 SD = 0.355 (individual)) and on* depression* (*F*(1) = 29.125; *p* = 0.000  *p* < 0.05; *η* = 0.227; Observed Potential = 1.000; *M* = 0.504 (group); SD = 0.423; *M* = 0.158 SD = 0.159 (individual)). This aspect has a very slight yet significant effect on the* quality of life* (*F*(1) = 3.778; *p* = 0.053  *p* < 0.05; *η* = 0.017; Observed Potential = 0.490; *M* = 0.478 SD = 0.474 (individual); *M* = 0.327 SD = 0.574 (group)).

It is also important to note that there are significant differences of both* anxiety* (*F*(10) = 14.256; *p* = 0.000  *p* < 0.05; *η* = 0.506; Observed Potential = 1.000) and* quality of life* (*F*(13) = 8.995; *p* = 0.000  *p* < 0.05; *η* = 0.363; Observed Potential = 1.000) effect sizes among the* instruments* adopted.

There is a positive and high correlation between the* number of sessions* of relaxation techniques and the effect sizes of FEV_1_ (*X* ^2^(9) = 90.000; *p* = 0.000 for *p* < 0.05), indicating that a more constant practice can improve the FEV_1_. In addition, there is a similar connection between the number of sessions and the* anxiety*'s effect size (*X* ^2^(35) = 280.000; *p* = 0.000 for *p* < 0.05), depression's effect size (*X* ^2^(40) = 290.000; *p* = 0.000 for *p* < 0.05), and the* quality of life*'s effect size (*X* ^2^(135) = 1278.000; *p* = 0.000 for *p* < 0.05). Regarding the* minutes per session*, there are relevant correlations with the effect size about* anxiety* (*r* = −0.487; *p* = 0.000 for *p* < 0.05), depression (*r* = −0.637; *p* = 0.000 for *p* < 0.05), and* quality of life* (*r* = −0.217; *p* = 0.002 for *p* < 0.05). These correlations are negative, which means that the improvement of one variable such as the relaxation time is inversely related to the other (e.g., anxiety). There are no other relevant differences.

## 4. Discussion

This meta-analysis evaluated the effects of the relaxation training on Forced Expiratory Volume in the First Second (FEV_1_), anxiety, depression, and quality of life of people with COPD.

The effect sizes concerning all the examined variables are positive, but they reach only Cohen's “small effect.” There is a high heterogeneity between studies, together with a low stability, probably because the studies included in our analysis are few and not all of them analyze each of the variables considered in this research. Moreover, a high level of heterogeneity may be an indication that the overall effectiveness of the treatment can be attributed to all interventions related to the application of relaxation techniques, nevertheless having specific characteristics and different methods of application and using various assessment instruments. Precisely for these reasons, the results should be interpreted with caution. Given our findings, it can be argued that health-care professionals should focus on particular variables in the application of the relaxation trainings and in particular the setting or therapeutic relationship rather than what patients perceive as particularly suited to meet their needs, such as information about their care.

Furthermore, other meta-analyses concerning relaxation techniques for the management of COPD cannot be found in literature; consequently, this could constitute an advantage of this analysis. Effectively, previous systematic studies and meta-analyses investigated the efficacy of cognitive behavioral or psychotherapeutically based interventions and progressive muscle relaxation, though not distinguishing them [16]. Others examined the effects of education, exercise, and/or psychosocial support [65] in COPD patients, the effects of psychologically based treatments only on anxiety and panic in people with COPD [66], or the improvements in respiratory functions in people with Cystic Fibrosis (CF) generated by meditative movements [67]. Finally, other authors investigated the effectiveness of nonpharmacological and noninvasive interventions to relieve breathlessness in participants with advanced stages of cancer, Chronic Obstructive Pulmonary Disease (COPD), interstitial lung disease, chronic heart failure, or motor neuron disease [68]. Therefore, despite the fact that our meta-analysis did not produce striking results, the study could be a valuable tool for increasing the knowledge about a more effective application of relaxation techniques in COPD patients. However, it is also important to note that, in addition to the limits discussed in this paragraph, we should pay attention to the fact that some criteria were very specific, which can be a strength but also a limitation with respect to the inclusion of other studies.

Using a between-group analysis it was possible to outline the effects produced by these techniques and the effects derived from the time of the application of usual care. One of the most obvious benefits is that the meta-analysis allows us to integrate the research literature relating to the same subject, beyond the limitations of each study considered separately. In addition, using data from different studies, it is possible to increase the accuracy compared to the estimate of the treatment efficacy and detect effects previously latent, since the individual studies are characterized by a low statistical weight. Moreover, the presence of meta-analytical studies in literature should encourage researchers to proceed in a methodologically rigorous way in conducting their experiments, just to greatly increase the odds of their inclusion [69]. However, in this as well as in other meta-analyses, there appears to be a limit, that is, the need for the pooled studies to have very similar characteristics in order to avoid false conclusions from certain intervening variables which have not been attended to or considered influential [70]. Furthermore, it is important to note that it is difficult to derive conclusions from effect sizes on real patients; indeed, the data examined in meta-analysis tend to focus on a subject “average,” which is eligible for part of a research design. It is also important to note that many of the studies did not consider all the variables examined as moderators of the effect, an aspect that can significantly reduce the stability of the same meta-analysis and therefore the ability to draw firm conclusions. Moreover, there is also a limit to the generalizability of results, because we restricted the search to some computerized databases and we adopted only RCT studies as inclusion criteria. Moreover, the number of studies considered is small, due to the fact that in the literature many studies did not report essential information for computing the effect sizes. Consequently, it is necessary to be cautious when interpreting the results, especially when there is low heterogeneity detected between studies, as well as the low stability of the analysis.

These considerations may therefore have important implications for future studies; from a theoretical point of view, they might be useful for identifying new factors that moderate the effects of various dimensions, while, from a methodological point of view, they would be useful for adopting measurements, that is, more stringent and sampling techniques, but most importantly control strategies. Moreover, despite the fact that this meta-analysis has failed to reveal significant effects of the implementation of relaxation techniques for COPD management, it can be a starting point for understanding how to increase the quality of the proposed interventions, pointing out that many studies that have been considered separately demonstrated some sort of efficacy.

## 5. Conclusions

Even if this meta-analysis is not able to reveal the effectiveness of relaxation techniques, it is important to remind ourselves that there are previous studies which have demonstrated that these trainings sessions could decrease anxiety and psychological distress and produce benefits for some physiological parameters such as oxygen saturation and heart rate not exclusively in people with COPD [71–73]. Future studies are necessary, while taking methodological precautions such as paying attention to the sampling techniques, measurements, confounding variables, and control strategies.

## Figures and Tables

**Table 1:** Inclusion and exclusion criteria.

Category	Criteria
Study population	Individuals hospitalized or outpatients
	Patients with Chronic Obstructive Pulmonary Disease (COPD) or with severe airflow obstruction

Time period	1970–2014

Publication languages	English

Admissible study designs	Randomized Controlled Trial (RCT)
	Studies that provide sufficient detail regarding methods and results to enable use and adjustment of the data to effect size computation

Interventions	Must approach one or more of the following interventions: (i) relaxation techniques; (ii) progressive muscle relaxation; (iii) guided imagery; (iv) distraction therapy; (v) biofeedback; (vi) breathing techniques (diaphragmatic breathing, Pursed-Lips Breathing); (vii) yoga; (viii) Tai Chi; (ix) acupressure
	Not allowed studies providing for the comparison between a control group and one subjected to a pulmonary rehabilitation in which the relaxation constituted only one component of a few minutes
	Not allowed studies that compared two rehabilitation programs in which relaxation was present equally

Control group	Patients with Chronic Obstructive Pulmonary Disease (COPD), hospitalized or outpatients
	Not allowed studies with healthy subjects and volunteers or with other diseases in the control group
	It has not been subjected to any treatment or to usual rehabilitation treatments or placebo or to activities not expressly relaxing (i.e., handcrafts)

Variables	Must assess baseline and outcome data for one or more of the following variables: (i) percentage of Forced Expiratory Volume in One Second (FEV_1_); (ii) anxiety; (iii) depression; (iv) quality of life (QoL)

Other information	If possible, they should provide data on other important variables, comprising those in text and tables: (i) number of subjects; (ii) mean age; (iii) geographic origin; (iv) assessment measures; (v) homework; (vi) number of session or protocol length; (vii) trial context.

**Table 2:** Characteristics of the studies included.

Characteristics of the studies																								
Title	References	Country	Year	Study design	Statistical analysis	Type of training	Control group activity	Type of subjects	Pulmonary functioning	Instrument	Individual/group	Homework	Duration	*N* ^∧^ of sessions	*N* ^∧^ subjects	*N* ^∧^ experimental subjects	*N* ^∧^ control subjects	*N* ^∧^ third group	Dropout	*N* ^∧^ female	*N* ^∧^ male	Mean age	SD age	Quality
The Effects of Controlled Breathing during Pulmonary Rehabilitation in Patients with COPD	[32]	Germany	2011	RCT	Within-groups comparisons: paired Student's *t*-tests; between groups: unpaired Student's *t*-tests	RBF (Respiratory Biofeedback Training) and breathing techniques	Physical exercise training only	COPD patients	GOLD 1-2-3-4	CRQ	Individual	No	3-4 weeks	10 sessions	40	20	20	0	Nd	23	17	66.1	6.4	6

Can Ventilation-Feedback Training Augment Exercise Tolerance in Patients with COPD?	[37]	USA	2007-2008	RCT	Analysis of covariance, paired *t*-tests	Ventilation Biofeedback Training	Exercise alone	COPD patients	GOLD 3	CRQ	Individual	No	12 weeks	36 sessions	64	22	20	20	15	Nd	Nd	67.3		6

Tai Chi Exercise for Patients with Chronic Obstructive Pulmonary Disease: A Pilot Study	[29]	USA	2010	RCT	*t*-tests for continuous variables and Fisher's exact test for nominal variables, 2-sample Wilcoxon rank-sum tests	Tai Chi and breathing exercises	Usual care alone	COPD patients	GOLD 2	CRQ; CES-D	Group	Yes	12 weeks	24 sessions	10	5	5	0	0	4	6	66	6	7

Exercise Maintenance following Pulmonary Rehabilitation Effect of Distractive Stimuli	[34]	USA	2002	RCT	2*∗*3 multivariate analysis of variance, 2*∗*3 multivariate analysis of variance	DAS (Distractive Auditory Stimuli)	Walk at their own pace from 20 to 45 minutes	COPD patients	GOLD 2	STAI; CES-D; SGRQ; VAS (global QoL)	Group	No	4 weeks	From 2 to 5 times a week	24	12	12	0	0	20	4	68.1	8	6

Distraction Therapy with Nature Sights and Sounds Reduces Pain during Flexible Bronchoscopy: A Complementary Approach to Routine Analgesia	[38]	USA	2003	RCT	Chi-square test and Student's *t*-test, ordinal logistic regression	Distraction therapy	Usual care	COPD and asthma patients, with FB (Flexible Bronchoscopy)	Nd	STAI	Individual	No	4 months	Nd	80	41	39	0	0	42	38	53.8		6

Effects of Progressive Muscle Relaxation Training on Anxiety and Depression in Patients Enrolled in an Outpatient Pulmonary Rehabilitation Program	[21]	USA	2008	Prospective RCT	Independent *t*-test for continuous variables and 2 tests for categorical variables, 2-factor repeated measures analysis of variance test	Progressive muscle relaxation	Standard PR program (exercise training, education, and psychosocial support) without PMR	COPD patients	GOLD 2	HADS	Individual	No	8 weeks	8 sessions	103	37	46	0	20	Nd	Nd	65.5		6

Treatment of Dyspnea in COPD: A Controlled Clinical Trial of Dyspnea Management Strategies	[39]	USA	1995	RCT	2*∗*3 analysis of variance with repeated measures, Greenhouse-Geisser adjusted degrees of freedom, independent *t*-tests	Progressive muscle relaxation, breathing retraining, pacing, self-talk and panic control	General health education	COPD patients	GOLD 2-3	STAI; CES-D; QWB	Group	No	6 weeks	6 sessions	98	46	43	0	9	40	49	67.4		7

Efficacy of Pursed-Lips Breathing: A Breathing Pattern Retraining Strategy for Dyspnea Reduction	[40]	USA	2007	RCT	Analysis of variance, multilevel modeling	PLB (Pursed-Lips Breathing)	Usual care	COPD patients	GOLD 3	SF-36	Individual	No	12 weeks	Nd	40	14	13	13	0	2	38	65	9	7

Diaphragmatic Breathing Training Program Improves Abdominal Motion during Natural Breathing in Patients with Chronic Obstructive Pulmonary Disease: A Randomized Controlled Trial	[41]	Brazil	2012	Prospective RCT, single blind	Independent *t*-test, chi-square test, analysis of covariance, Pearson's correlation test	Diaphragmatic breathing	Usual care	COPD patients	GOLD 3	SGRQ	Individual	No	4 weeks	12 sessions	30	15	15	0	0	8	22	66.4		7

Effects of Singing Classes on Pulmonary Function and Quality of Life of COPD Patients	[42]	Brazil	2009	RCT	Student's *t*-test, chi-square test	Breathing techniques and vocalization	Handcraft work	COPD patients	GOLD 2-3	SGRQ	Group	No	24 weeks	24 sessions	16	15	15	0	13 (disc)	6	24	71.7		6

Singing Teaching as a Therapy for Chronic Respiratory Disease: A RCT and Qualitative Evaluation	[43]	UK	2010	RCT	*t*-tests	Relaxation, vocalization, posture	Usual care	COPD patients	GOLD 3	HADS; SGRQ; SF-36	Individual	Yes	6 weeks	12 sessions	36	15	13	0	8 (follow-up)	Nd	Nd	67.3	8.1	5

Yoga Therapy Decreases Dyspnea-Related Distress and Improves Functional Performance in People with Chronic Obstructive Pulmonary Disease: A Pilot Study	[24]	USA	2009	RCT	Two-way repeated measures analysis of variance	Yoga	Usual care	COPD patients	GOLD 2-3	SF-36; SSAI; CES-D; CRQ	Group	Yes	12 weeks	24 sessions	29	14	15	0	0	21	8	69.9	9.5	6

Effectiveness of a Tai Chi Qigong Program in Promoting Health-Related Quality of Life and Perceived Social Support in Chronic Obstructive Pulmonary Disease Clients	[44]	China	2010	RCT, single blind	Repeated measures analysis of variance (RANOVA)	Tai Chi Qigong	Usual care	COPD patients	GOLD 1-2-3	SGRQ	Group	Yes	12 weeks	24 sessions	206	70	67	69	48	18	188	72.9		6

Functional and Psychosocial Effects of Health Qigong in Patients with COPD: A Randomized Controlled Trial	[45]	China	2010-2011	RCT	Intention-to-treat (ITT) analysis, Student's *t*-test and Fisher exact test, repeated measures analysis of variance (ANOVA)	Tai Chi Qigong	Training sessions reinforcing the breathing and walking exercise	COPD patients	GOLD 3	SF-36; Chinese CRQ	Individual	Yes	6 months	from 1 to 4 times a day	80	23	29	0	19	9	71	72.4		8

Singing Classes for Chronic Obstructive Pulmonary Disease: A Randomized Controlled Trial	[46]	UK	2012	RCT	ANCOVA	Breathing techniques, relaxation training, vocalization	Film workshops	COPD patients	GOLD 3	HADS; SF-36	Group	Yes	8 weeks	16 sessions	33	13	11	0	8	Nd	Nd	68.4		6

Role of Pranayama in Rehabilitation of COPD Patients: A Randomized Controlled Study	[25]	India	2006	Prospective RCT	Student Newman-Keuls tests	Yoga	Usual physical activity	COPD patients	GOLD 2-3	SGRQ	Group	No	3 months	Half an hour, everyday	48	23	22	0	3	7	38	52.2		5

Adjunct Treatment with Yoga in Chronic Severe Airways Obstruction	[47]	Australia	1978	RCT	Student's *t*-test	Yoga	Physiotherapy	Patients with severe airways obstruction, with or without emphysema	GOLD 3	Only FEV_1_	Group	Nd	9 months	3 times a week for the first four weeks, 2 for the other four, and 1 for the remaining	24	12	12	0	0	Nd	Nd	60		4

Pulmonary Rehabilitation Improves Depression, Anxiety, Dyspnea, and Health Status in Patients with COPD	[48]	USA	2007	RCT	*t*-test for independent and dependent samples; Pearson's correlation test	Relaxation, breathing techniques, conservation of energy	Usual care	COPD patients	GOLD 3	STAI; SGRQ; BDI	Group	No	8 weeks	24 sessions	24	10	14	0	0	6	18	64.5		5

Effectiveness of Incorporating Tai Chi in Pulmonary Rehabilitation Program for Patients in Primary Health Care (COPD)	[49]	China	2011–2013	Prospective RCT, single blind	ANCOVA	Tai Chi and relaxation exercises	Pulmonary rehabilitation without Tai-Chi	COPD patients	GOLD 2	SGRQ	Group	No	6 months	Nd	192	94	98	0	28 (follow-up) 26 (disc)	17	175	74	6.624	5

Short-Form Sun-Style Tai Chi as an Exercise Training Modality in People with COPD	[50]	Australia	2010–2012	RCT	Paired *t*-tests and repeated measures analysis of variance with intention-to-treat analysis	Sun-Style Tai Chi and breathing exercises	Usual medical care	COPD patients	GOLD 2	CRQ; HADS	Group	Yes	3 months	12 sessions	42	19	19	0	4	15	27	73	8	6

Relaxation to Reduce Dyspnea and Anxiety in COPD Patients	[51]	USA	1992	RCT	ANOVA	Progressive muscle relaxation	Sit quietly	COPD patients	GOLD 2	STAI	Individual	Yes	Nd	3-4 times a week	26	13	13	0	8	Nd	Nd	67		4

One-Session Cognitive Behavioural Therapy for Elderly Patients with Chronic Obstructive Pulmonary Disease	[52]	USA	2001	RCT, single blind	*t*-test, chi-square, MANOVA	Relaxation training and breathing techniques	2 h of COPD education, followed by weekly calls	COPD patients	Nd	GDS; BAI; SF-36	Group	Yes	6 weeks	1 session, followed by daily sessions autonomously	53	21	27	0	5	9	44	71.3	5.9	4

Effects of Psychotherapy in Moderately Severe COPD: A Pilot Study	[53]	UK	1997	RCT	Paired *t*-tests, unpaired tests	Muscle relaxation, breathing technique, distraction therapy	They attended the laboratory for seven times, usual visits	COPD patients	GOLD 2-3-4	HADS; SGRQ	Group	Yes	6 weeks	6 sessions	18	10	8	0	0	10	8	72.2		3

Comparison of the Effectiveness of Music and Progressive Muscle Relaxation for Anxiety in COPD: A Randomized Controlled Pilot Study	[54]	India	2009	RCT	ANOVA	Progressive muscle relaxation (PMR)	Music	COPD patients	Nd	STAI	Individual	Nd	1 day	2 sessions	72	32	32	0	8	19	45	63	7.5	6

Relax and Breathe Deeply: A Quick Relaxation Training for People with Chronic Obstructive Pulmonary Disease	[55], [submitted]	Italy	2015	RCT, single blind	Between-groups comparisons: Mann-Whitney *U* test; within groups comparisons: Wilcoxon test	Relaxation training and breathing techniques	They watched a documentary movie	COPD patients	GOLD 2-3	VAS; STAI; PANAS; Short FSS	Individual	No	1 day	1 session	38	19	19	0	0	15	23	72.66	8.68	7

**Table 3:** Effect sizes of each study included.

Studies	Effect size: Time 2 (8–12 months)			
	Effect size BT %FEV_1_	Effect size BT anxiety	Effect size BT depression	Effect size BT QoL
The Effects of Controlled Breathing during Pulmonary Rehabilitation in Patients with COPD [32]	0.146			0.397
Can Ventilation-Feedback Training Augment Exercise Tolerance in Patients with COPD? [37]				0.527
VF Alone versus Exercise Alone [37]				0.540
Tai Chi Exercise for Patients with Chronic Obstructive Pulmonary Disease: A Pilot Study [29]			0.804	1.886
Exercise Maintenance following Pulmonary Rehabilitation Effect of Distractive Stimuli [34]		0.725	0.512	0.089
Distraction Therapy with Nature Sights and Sounds Reduces Pain during Flexible Bronchoscopy: A Complementary Approach to Routine Analgesia [38]		−0.133		
Effects of Progressive Muscle Relaxation Training on Anxiety and Depression in Patients Enrolled in an Outpatient Pulmonary Rehabilitation Program [21]		0.014	0.023	
Treatment of Dyspnea in COPD: A Controlled Clinical Trial of Dyspnea Management Strategies [39]		0.114	0.178	0.000
Efficacy of Pursed-Lips Breathing: A Breathing Pattern Retraining Strategy for Dyspnea Reduction [40]				0.281
EMT versus Control [40]				0.234
Diaphragmatic Breathing Training Program Improves Abdominal Motion during Natural Breathing in Patients with Chronic Obstructive Pulmonary Disease: A Randomized Controlled Trial [41]	0.050			1.137
Effects of Singing Classes on Pulmonary Function and Quality of Life of COPD Patients [42]				1.484
Singing Teaching as a Therapy for Chronic Respiratory Disease: A RCT and Qualitative Evaluation [43]		0.392	0.507	0.323
Yoga Therapy Decreases Dyspnea-Related Distress and Improves Functional Performance in People with Chronic Obstructive Pulmonary Disease: A Pilot Study [24]	0.325	0.134	0.246	0.151
Effectiveness of a Tai Chi Qigong Program in Promoting Health-Related Quality of Life and Perceived Social Support in Chronic Obstructive Pulmonary Disease Clients [44]				0.019
Exercise Group versus Control Group [44]				0.140
Functional and Psychosocial Effects of Health Qigong in Patients with COPD: A Randomized Controlled Trial [45]				1.333
Singing Classes for Chronic Obstructive Pulmonary Disease: A Randomized Controlled Trial [46]		0.110	0.708	0.099
Role of Pranayama in Rehabilitation of COPD Patients: A Randomized Controlled Study [25]	1.950			2.174
Adjunct Treatment with Yoga in Chronic Severe Airways Obstruction [47]	0.145			
Pulmonary Rehabilitation Improves Depression, Anxiety, Dyspnea, and Health Status in Patients with COPD [48]		0.790	1.169	0.897
Effectiveness of Incorporating Tai Chi in Pulmonary Rehabilitation Program for Chronic Obstructive Pulmonary Disease Patients in Primary Health Care (COPD) [49]	−0.140			0.102
Short-Form Sun-Style Tai Chi as an Exercise Training Modality in People with COPD [50]		0.632	0.283	0.527
Relaxation to Reduce Dyspnea and Anxiety in COPD Patients [51]		1.154		
One-Session Cognitive Behavioural Therapy for Elderly Patients with Chronic Obstructive Pulmonary Disease [52]		0.086	0.084	−0.029
Effects of Psychotherapy in Moderately Severe COPD: A Pilot Study [53]		1.414	1.414	0.824
Comparison of the Effectiveness of Music and Progressive Muscle Relaxation for Anxiety in COPD: A Randomized Controlled Pilot Study [54]		0.042		
Relax and Breathe Deeply: A Quick Relaxation Training for People with Chronic Obstructive Pulmonary Disease [55] [submitted]	0.678	0.821		
